# The Future of Luteal Phase Support in ART and the Role of Dydrogesterone

**DOI:** 10.3389/frph.2020.618838

**Published:** 2021-01-20

**Authors:** Panagiotis Drakopoulos, Caroline Roelens, Michel De Vos, Shari Mackens, AnnaLisa Racca, Herman Tournaye, Christophe Blockeel

**Affiliations:** ^1^Center for Reproductive Medicine, Universitair Ziekenhuis Brussel, Vrije Universiteit Brussel, Brussels, Belgium; ^2^Department of Obstetrics, Gynecology, Perinatology and Reproductology, Institute of Professional Education, Sechenov University, Moscow, Russia; ^3^Department of Obstetrics and Gynaecology, University of Zagreb, Zagreb, Croatia

**Keywords:** luteal (phase) support, dydrogesterone, progesterone, endometrium, IVF

## Introduction

*In vitro* fertilization (IVF) treatment routinely involves ovarian stimulation (OS) with gonadotropins in combination with GnRH analogs to prevent premature luteinization and ovulation (1). However, it is well-established that the use of GnRH analogs during OS may impair corpus luteum function, resulting in suboptimal endometrial receptivity (2). Thus, luteal phase support (LPS) with progestins is an essential part of IVF treatment and is mandatory to support implantation and to increase pregnancy rates after fresh embryo transfer (3).

## Type of Progesterone and Route of Administration

Progestins can be administered using various routes, either vaginally, intramuscularly (IM), rectally, orally, or subcutaneously, with differential impact on the pharmacokinetics of progestins. Indeed, serum progesterone (P) levels are higher when progestins are administered using the IM route compared to the vaginal one. On the other hand, although micronized P capsules had initially been developed for oral use, they can be administered vaginally, offering an effective alternative to oral and IM injections: serum P concentrations may be lower after vaginal compared to IM administration, but endometrial P levels are higher because of the uterine first pass effect, while several disadvantages of the IM route (i.e., injection site pain and discomfort, risk of infection) are avoided (4). On a similar note, vaginal micronized P is preferred over oral administration due to the rapid absorption and avoidance of the first-pass metabolism (5, 6). However, there are a number of downsides to the vaginal route of P administration, since vaginal irritation, discharge and bleeding may occur (7). Furthermore, suboptimal serum P levels in a subgroup of women who are prescribed vaginal P may be associated with reduced pregnancy rates (8). Therefore, vaginal micronized P administration should not be seen as a panacea.

In this regard, the concept of oral progestin administration in assisted reproduction technology (ART) has recently been revitalized, given that dydrogesterone (6-dehydro-retroprogesterone) has been extensively used for the treatment of other conditions associated with P deficiency since the 1960s (9). Dydrogesterone is a stereo-isomer of P, with an additional double bond between carbons 6 and 7, characterized by a better oral bioavailability and higher specificity for P receptors compared with oral micronized P (10, 11). A recent study demonstrated that after natural conception dydrogesterone supplementation from 6 to 20 weeks of gestation significantly reduced the incidence of preeclampsia (PE) in high-risk patients (12), while these findings were replicated by another retrospective study showing a reduction in PE rate after dydrogesterone supplementation in assisted reproductive techniques and intra-uterine insemination (13).

### Clinical Studies Evaluating Dydrogesterone in Fresh Cycle IVF

Several small-scale clinical studies have shown that oral dydrogesterone is at least as efficacious as micronized vaginal progesterone in supporting pregnancy following fresh embryo transfer (14–16). These findings revived the interest in oral dydrogesterone for LPS and paved the way for large Phase III prospective RCTs (Lotus I and Lotus II studies), which led to the recent approval of oral dydrogesterone for LPS in IVF–ART.

In particular, Lotus I was an international Phase III non-inferiority RCT including 1,034 patients undergoing IVF and fresh embryo transfer, which showed that dydrogesterone 30 mg (10 mg three times daily) resulted in comparable ongoing pregnancy rates (pregnancy rates at 12 weeks of gestation of 37.6 and 33.1% in the oral dydrogesterone and micronized vaginal P group, respectively) compared to vaginal micronized P 600 mg (200 mg three times daily) (17). Similarly, Lotus II RCT compared oral dydrogesterone 30 mg (10 mg three times daily) with 8% micronized vaginal P gel (90 mg once daily) and demonstrated non-inferiority, with ongoing pregnancy rates at 12 weeks' gestation of 38.7% in the oral dydrogesterone group and 35.0% in the micronized vaginal progesterone gel group (18). The main conclusion of the two RCTs was that oral dydrogesterone is safe (no evidence for an increased risk for fetal malformation), well-tolerated and as efficient as vaginal P.

### Clinical Studies Evaluating Dydrogesterone in Frozen Embryo Transfer Cycles

Frozen-thawed embryo transfer (FET) has become an increasingly important part of IVF treatment, with large clinical trials and meta-analyses demonstrating similar live birth rates to those associated with fresh embryo transfer (19). To date, several methods of endometrial preparation for FET have been developed, with hormone replacement therapy (HRT)-FET cycles being the most commonly used, in view of the reduced need for treatment monitoring and easier scheduling. In HRT-FET cycles estrogen and progesterone are administered consecutively, in order to mimic the endocrine conditions of the endometrium of a normal menstrual cycle. However, from an physiological point of view, LPS in HRT-FET is completely different compared to LPS in a fresh IVF cycle due to the lack of ovulation and absence of endogenous corpora lutea, suggesting that transformation of the endometrium into a receptive state for the implanting embryo is completely dependent on exogenous P supplementation (20).

While there is robust evidence demonstrating the efficacy of oral dydrogesterone for LPS in fresh IVF cycles as mentioned above, very few small studies using inconsistent doses have evaluated the role of dydrogesterone in HRT-FET cycles [(21–24); Table 1]. In the only RCT performed up to date, Zarei et al. (22) reported lower pregnancy rates in the oral dydrogesterone group compared to the micronized vaginal P group, using doses of 20 and 800 mg, respectively. However, the lack of data with regard to the optimal dosing of oral dydrogesterone in FET-HRT, highlights the need for further studies. In view of the advancing understanding of the impact of an absent corpus luteum in FET-HRT cycles and the associated elevated risk for PE (25), dydrogesterone with its potential immunomodulatory effects (26) represents an interesting research track. Of great importance will be the development of a clinically applicable dose monitoring test for dydrogesterone and/or its metabolites, as an optimal LPS presumably lies in its individualization (8).

**Table 1:** Overview of evidence of dydrogesterone use in HRT-FET cycles.

**Study**	***N***	**Study design**	**LPS in HRT-FET**	**Embryo stage**	**Outcome**
Zarei et al. (22)	400	RCT	400 mg MVP 2x/d vs. 10 mg DYD 2x/d vs. 10 mg DYD 2x/d + 0.1 mg GnRHa vs. 10 mg DYD 2x/day + 1500 IU hCG	Cleavage stage	CPR 20, 9, 25, and 17% (*p* = 0.03) OPR 18, 9, 3, and 17% (*p* = 0.07) MR 18.1, 35.7, 14.8, and 19.1% (*p* = 0.84)
Alahmad et al. (24)	314	Retrospective	MVP 600 mg/day of 90 mg vs. DYD 10 mg 3x/day	2PN	Cumulative CPR Difference: 1.4%, 95% CI: (−9.4 to 12.6), *p* = 0.80 CPR of first FET Difference: −3.2%, 95%CI: (−12.8 to 7.4), *p* = 0.54
Guo et al. (21)	529	Retrospective	DYD 10 mg 4x/day vs. IM P4 60 mg/day	Cleavage stage/blastocyst	CPR IR MR EPR OPR DR no significant difference
Rashidi et al. (23)	180	Pilot RCT	IM P4 50 mg 2x/d vs. DYD 20 mg 2x/d vs. MVP 400 mg 2x/d	95% cleavage stage5% blastcyst	CPR MR LBR no significant difference

*LPS, luteal phase support; HRT, hormone replacement therapy; FET, frozen embryo transfer; RCT, randomized controlled trial; MVP, micronized vaginal progesterone, DYD, dydrogesterone; GnRHa, gonadotrophin releasing hormone agonist; hCG, human chorionic gonadotrophin; IM P4, intramuscular progesterone; PN, pronuclei; CPR, clinical pregnancy rate; OPR, ongoing pregnancy rate; MR, miscarriage rate; IR, implantation rate; EPR, ectopic pregnancy rate; DR, delivery rate; LBR, life birth rate*.

## Conclusion

Collectively, there is evidence that dydrogesterone has high oral bioavailability and specificity for P receptors (27), suggesting that it is effective at a dose 10–20 times lower than that of micronized P (25). Dydrogesterone has a good safety and tolerability profile with few side effects, making the ideal candidate for LPS in ART. Oral dydrogestrone is as effective as vaginal progesterone for LPS in women undergoing fresh IVF, whilst more evidence is warranted for its use in HRT cycles. The well-known widespread preference of women for an oral compound may pave the way for dydrogesterone to become the new standard. Furthermore, preliminary observations showing a decreased risk of PE after dydrogesterone supplementation in natural and intrauterine insemination cycles (12, 13) may be of paramount value in HRT-FET cycles, which are known to have a higher incidence of PE (28).

## Author Contributions

All authors contributed to this work and approved the final version.

## Conflict of Interest

The authors declare that the research was conducted in the absence of any commercial or financial relationships that could be construed as a potential conflict of interest.

## References

[1] Ovarian Stimulation, T Bosch E Broer S Griesinger G Grynberg M Humaidan P. ESHRE guideline: ovarian stimulation for IVF/ICSI(dagger). Hum Reprod Open. (2020) 2020:hoaa009. 10.1093/hropen/hoaa009 32395637 PMC7203749

[2] Beckers NG Laven JS Eijkemans MJ Fauser BC. Follicular and luteal phase characteristics following early cessation of gonadotrophin-releasing hormone agonist during ovarian stimulation for in-vitro fertilization. Hum Reproduct. (2000) 15:43–9. 10.1093/humrep/15.1.43 10611186

[3] Delcour C Robin G Delesalle AS Drumez E Plouvier P Dewailly D. Weekly intramuscular progesterone for luteal phase support in women receiving oocyte donation is associated with a decreased miscarriage rate. Reproduct Biomed Online. (2019) 39:446–51. 10.1016/j.rbmo.2019.05.001 31311693

[4] Miles RA Paulson RJ Lobo RA Press MF Dahmoush L Sauer MV. Pharmacokinetics and endometrial tissue levels of progesterone after administration by intramuscular and vaginal routes: a comparative study. Fertility Sterility. (1994) 62:485–90. 10.1016/S0015-0282(16)56935-0 8062942

[5] Bulletti C de Ziegler D Flamigni C Giacomucci E Polli V Bolelli G. Targeted drug delivery in gynaecology: the first uterine pass effect. Hum Reproduct. (1997) 12:1073–9. 10.1093/humrep/12.5.1073 9194669

[6] Cicinelli E de Ziegler D. Transvaginal progesterone: evidence for a new functional 'portal system' flowing from the vagina to the uterus. Hum Reproduct Update. (1999) 5:365–72. 10.1093/humupd/5.4.365 10465526

[7] van der Linden M Buckingham K Farquhar C Kremer JA Metwally M. Luteal phase support for assisted reproduction cycles. Cochrane Database Syst Rev. (2015) 2015:CD009154. 10.1002/14651858.CD009154.pub3 26148507 PMC6461197

[8] Labarta E Mariani G Holtmann N Celada P Remohi J Bosch E. Low serum progesterone on the day of embryo transfer is associated with a diminished ongoing pregnancy rate in oocyte donation cycles after artificial endometrial preparation: a prospective study. Hum Reproduct. (2017) 32:2437–42. 10.1093/humrep/dex316 29040638

[9] Backer MH Jr. Isopregnenone (Duphaston): a new progestational agent. Obstet Gynecol. (1962). 19:724–9. 13863774

[10] Schindler AE. Progestational effects of dydrogesterone in vitro, in vivo and on the human endometrium. Maturitas. (2009) 65(Suppl. 1):S3–11. 10.1016/j.maturitas.2009.10.011 19969432

[11] Rizner TL Brozic P Doucette C Turek-Etienne T Muller-Vieira U Sonneveld E. Selectivity and potency of the retroprogesterone dydrogesterone *in vitro*. Steroids. (2011) 76:607–15. 10.1016/j.steroids.2011.02.043 21376746

[12] Tskhay V Schindler A Shestakova M Klimova O Narkevich capital A C. The role of progestogen supplementation (dydrogesterone) in the prevention of preeclampsia. Gynecol. Endocrinol. (2020) 36:698–701. 10.1080/09513590.2019.1706085 31876197

[13] Ali AB Ahmad MF Kwang NB Shan LP Shafie NM Omar MH. Dydrogesterone support following assisted reproductive technique (ART) reduces the risk of pre-eclampsia. Horm Mol Biol Clin Investig. (2016) 27:93–6. 10.1515/hmbci-2015-0063 26910749

[14] Ganesh A Chakravorty N Mukherjee R Goswami S Chaudhury K Chakravarty B. Comparison of oral dydrogestrone with progesterone gel and micronized progesterone for luteal support in 1,373 women undergoing in vitro fertilization: a randomized clinical study. Fertility Sterility. (2011) 95:1961–5. 10.1016/j.fertnstert.2011.01.148 21333984

[15] Chakravarty BN Shirazee HH Dam P Goswami SK Chatterjee R Ghosh S. Oral dydrogesterone vs. intravaginal micronised progesterone as luteal phase support in assisted reproductive technology (ART) cycles: results of a randomised study. J Steroid Biochem Mol Biol. (2005) 97:416–20. 10.1016/j.jsbmb.2005.08.012 16213136

[16] Tomic V Tomic J Klaic DZ Kasum M Kuna K. Oral dydrogesterone vs. vaginal progesterone gel in the luteal phase support: randomized controlled trial. Eur J Obstetr Gynecol Reproduct Biol. (2015) 186:49–53. 10.1016/j.ejogrb.2014.11.002 25622239

[17] Tournaye H Sukhikh GT Kahler E Griesinger G. A phase III randomized controlled trial comparing the efficacy, safety and tolerability of oral dydrogesterone vs. micronized vaginal progesterone for luteal support in in vitro fertilization. Hum Reproduct. (2017) 32:2152. 10.1093/humrep/dex266 PMC6790500 28938733

[18] Griesinger G Blockeel C Sukhikh GT Patki A Dhorepatil B Yang DZ. Oral dydrogesterone vs. intravaginal micronized progesterone gel for luteal phase support in IVF: a randomized clinical trial. Hum Reproduct. (2018) 33:2212–21. 10.1093/humrep/dey306 PMC6238366 30304457

[19] Roque M Haahr T Geber S Esteves SC Humaidan P. Fresh vs. elective frozen embryo transfer in IVF/ICSI cycles: a systematic review and meta-analysis of reproductive outcomes. Hum Reproduct Update. (2019) 25:2–14. 10.1093/humupd/dmy033 30388233

[20] Ghobara T Gelbaya TA Ayeleke RO. Cycle regimens for frozen-thawed embryo transfer. Cochrane Database Syst Rev. (2017) 7:CD003414. 10.1002/14651858.CD003414.pub3 28675921 PMC6483463

[21] Guo W Chen X Ye D He Y Li P Niu J. [Effects of oral dydrogesterone on clinical outcomes of frozen-thawed embryo transfer cycles]. Nan Fang Yi Ke Da Xue Xue Bao. (2013) 33:861–5. 23803198

[22] Zarei A Sohail P Parsanezhad ME Alborzi S Samsami A Azizi M. Comparison of four protocols for luteal phase support in frozen-thawed Embryo transfer cycles: a randomized clinical trial. Arch Gynecol Obstet. (2017) 295:239–46. 10.1007/s00404-016-4217-4 27761732

[23] Rashidi B Ghazizadeh M Tehrani Nejad E Bagheri M Gorginzadeh M. Oral dydrogesterone for luteal support in frozen-thawed embryo transfer artificial cycles: a pilot randomized controlled trial. Asian Pacific J Reproduct. (2016) 5:490–4. 10.1016/j.apjr.2016.10.002

[24] Alahmad A Neumann K Depenbusch M Schultze-Mosgau A Osterholz-Zaleski T Hajek J. Oral dydrogesterone vs. micronized vaginal progesterone for artificial frozen-thawed embryo transfer cycles. Hum Reproduct. (2019) 34:i442.

[25] Schindler AE Campagnoli C Druckmann R Huber J Pasqualini JR Schweppe KW. Classification and pharmacology of progestins. Maturitas. (2003) 46(Suppl. 1):S7–16. 10.1016/j.maturitas.2003.09.014 14670641

[26] Griesinger G Tournaye H Macklon N Petraglia F Arck P Blockeel C. Dydrogesterone: pharmacological profile and mechanism of action as luteal phase support in assisted reproduction. Reproduct Biomed Online. (2019) 38:249–59. 10.1016/j.rbmo.2018.11.017 30595525

[27] Stanczyk FZ Hapgood JP Winer S Mishell DR Jr. Progestogens used in postmenopausal hormone therapy: differences in their pharmacological properties, intracellular actions, and clinical effects. Endocr Rev. (2013). 34:171–208. 10.1210/er.2012-1008 23238854 PMC3610676

[28] von Versen-Hoynck F Schaub AM Chi YY Chiu KH Liu J Lingis M. Increased preeclampsia risk and reduced aortic compliance with *in vitro* fertilization cycles in the absence of a corpus luteum. Hypertension. (2019) 73:640–9. 10.1161/HYPERTENSIONAHA.118.12043 30636552 PMC6434532

